# Alpha‐Fetoprotein Is a Potential Biomarker for Pancreatic Ductal Adenocarcinoma (PDAC): A Case Report

**DOI:** 10.1002/cnr2.70547

**Published:** 2026-04-22

**Authors:** Kaiwen Zhong, Xiaoying Wu, Xiangling Lin, Maoyun Xie, Min Deng, Qiang Tao, Wei Qin, Songlin Peng

**Affiliations:** ^1^ Department of General Surgery, the Seventh Affiliated Hospital Sun Yat‐Sen University Shenzhen China; ^2^ Pathological Diagnosis Center, the Seventh Affiliated Hospital Sun Yat‐Sen University Shenzhen China; ^3^ Department of Hepatobiliary and Pancreatic Surgery, the Eighth Affiliated Hospital Sun Yat‐Sen University Shenzhen China

**Keywords:** alpha‐fetoprotein, carbohydrate antigen 19–9, pancreatic ductal adenocarcinoma, superior mesenteric vein, vascular reconstruction

## Abstract

**Introduction:**

Pancreatic ductal adenocarcinoma (PDAC) is often diagnosed at an advanced stage, with high rates of mortality and drug resistance. Carbohydrate antigen 19‐9 (CA19‐9) is a tumor biomarker for diagnosis and prognosis in PDAC. However, elevated alpha‐fetoprotein (AFP) is exceedingly rare in the PDAC patient.

**Case Presentation:**

Herein, we describe a 67‐year‐old woman who presented with elevated AFP before the diagnosis of PDAC. In March 2025, this patient who had elevated AFP and CA19‐9 levels and was presenting with jaundice, was diagnosed with PDAC. Subsequently, she underwent pancreaticoduodenectomy, partial resection of the superior mesenteric vein (SMV), and SMV reconstruction. After radical resection (R0), the levels of AFP and CA19‐9 were significantly decreased. Next, she was treated with adjuvant chemotherapy (gemcitabine plus capecitabine). After four cycles of treatment, the patient presented with diarrhea, weight loss, poor nutritional status, and other signs of poor tolerance to chemotherapy. Thus, the regimen was changed to pembrolizumab plus trametinib. Hepatic recurrence was detected at the 7‐month follow‐up. At 11 months after surgery, the patient developed a biliary tract infection and obstructive jaundice, and ultimately died.

**Conclusions:**

This case demonstrates that AFP may be a potential biomarker for PDAC, especially in the CA19‐9‐negative PDAC patient.

**Abbreviations**
ACC: acinar cell carcinoma
AFP: alpha‐fetoprotein
AI: artificial intelligence
CA19‐9: carbohydrate antigen 19‐9
CBD: common bile duct
CEA: carcino‐embryonic antigen
cfDNA: circulating free DNA
CT: computed tomography
EUS: endoscopic ultrasonography
GB: gallbladder
HAC: hepatoid adenocarcinoma
HCC: hepatocellular carcinoma
HE: Hematoxylin–eosin
MDT: multi‐disciplinary treatment
MRI: magnetic resonance imaging
NCCN: National Comprehensive Cancer Network
PDAC: pancreatic ductal adenocarcinoma
pNET: pancreatic neuroendocrine tumors
POA: pancreatic oncofoetal antigen
PV: portal vein
SMV: superior mesenteric vein
SV: splenic vein

## 1 Introduction

Pancreatic ductal adenocarcinoma (PDAC) is a relatively rare cancer among all cancers, but its incidence increases by 0.5%–1.0% annually. Most patients present with locally advanced (30%–35%) or metastatic (50%–55%) disease at the time of diagnosis [1]. Currently, there are no high‐value serum biomarkers for screening pancreatic cancer. Elevated serum carbohydrate antigen 19‐9 (CA19‐9) level is observed in most pancreatic cancer patients, and CA19‐9 also helps to diagnose pancreatic cancer and predict prognosis. However, CA19‐9 lacks sufficient sensitivity and specificity for early detection, as it typically elevates only in advanced disease stages, limiting its utility for early PDAC screening [2]. In 2024, Ben‐Ami et al. indicated that CA19‐9 combined with TIMP1 and pancreatic‐specific methylated circulating free DNA (cfDNA) significantly enhances the detection efficiency of early‐stage PDAC [3]. Furthermore, compared to CA19‐9, biomarkers including microRNAs and circulating tumor DNA (ctDNA) multi‐omics combined with artificial intelligence (AI)‐assisted approaches are used to detect early pancreatic cancer, exhibiting higher specificity and sensitivity [4]. Strikingly, although multi‐omics approaches and AI‐assisted technologies require large, well‐annotated datasets to perform meaningful and unbiased analyses, these approaches have been quickly emerging into clinical practice.

Alpha‐fetoprotein (AFP) is commonly used as an adjunctive diagnostic tool and prognostic indicator for hepatocellular carcinoma (HCC) and genital system tumors, but it is not used as a serum marker for pancreatic‐related diseases. Previous literature reports indicated that pancreatic cancer, primarily including pancreatic neuroendocrine tumors (pNET), acinar cell carcinoma (ACC), pancreatoblastoma, and certain pancreatic cancers exhibiting hepatocellular differentiation, are associated with elevated AFP [5, 6, 7, 8, 9, 10, 11, 12]. However, PDAC with elevated AFP is extremely rare. A review of the literature revealed two documented cases of definitively diagnosed PDAC presenting with elevated AFP [13, 14]. The uniqueness of this case lies in the patient's persistently elevated AFP levels over about a 2‐year asymptomatic period without any positive clinical findings or symptoms. Herein, our case provides additional evidence that PDAC also possesses the capacity to produce AFP. This case underscores the importance of considering pancreatic cancer as a potential diagnosis when AFP levels are elevated in the absence of evidence for HCC and genital system tumors.

## 2 Case Presentation

**FIGURE 1:**
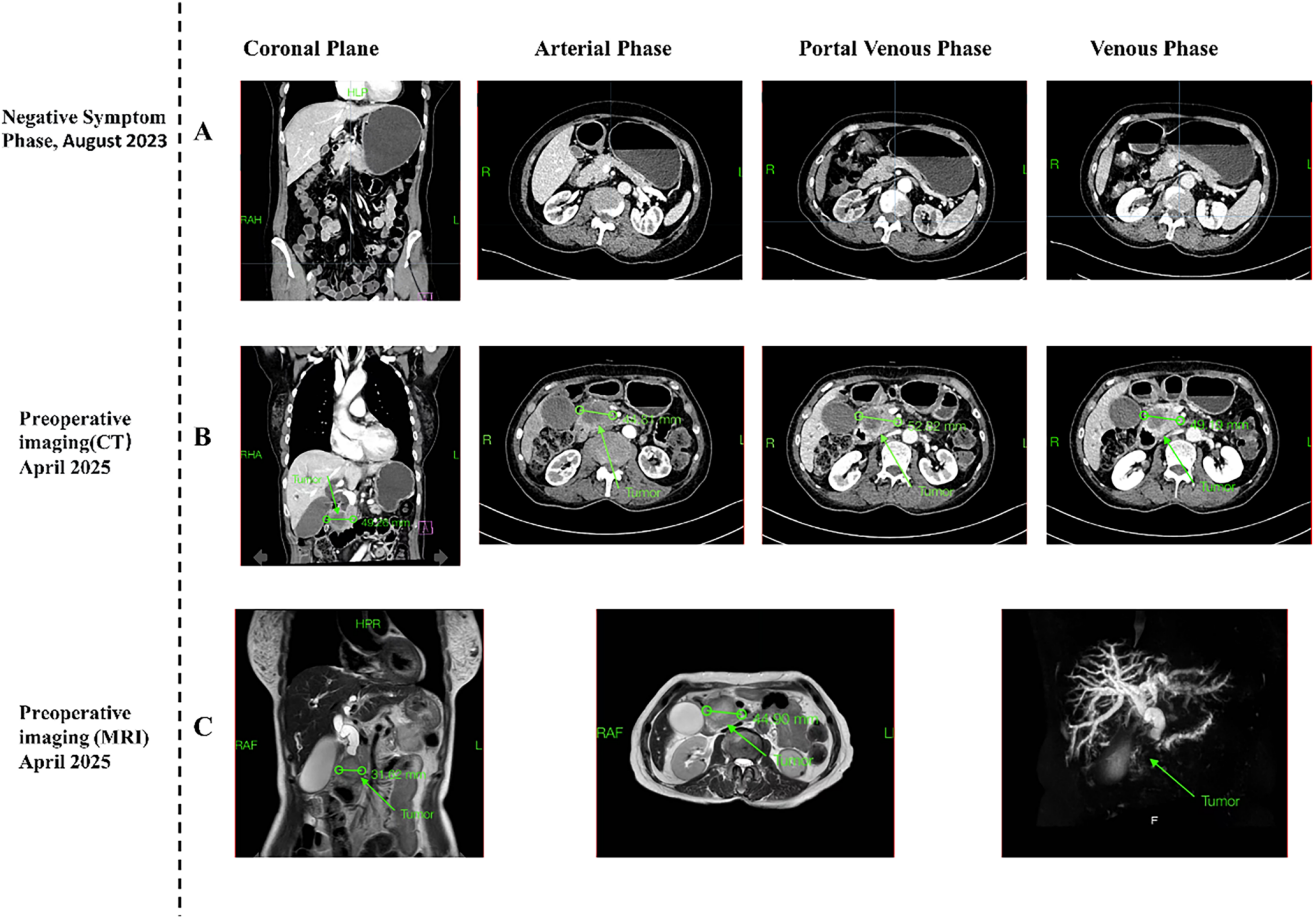
Disease progression of the patient. (A) In August 2023, contrast‐enhanced CT showed no abnormalities in the liver and pancreas. (B) In April 2025, preoperative contrast‐enhanced CT demonstrated a 4.0 × 5.0 cm mass in the pancreatic head with central non‐enhancing necrosis and peripheral ring enhancement, accompanied by gallbladder distension (Red arrows), but no hepatic lesions. (C) The lesion shows hyperintensity on T2‐weighted images with slightly irregular walls, no *liver metastasis*, bile ducts and the pancreatic duct dilation. Red arrows indicate a 4.8 × 4.8 cm tumor.

**FIGURE 2:**
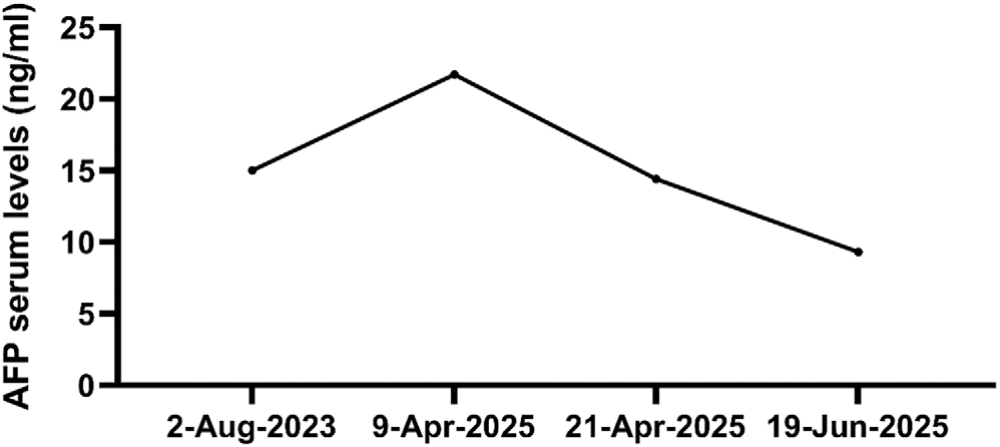
The changes of serum AFP levels. The AFP level was dramatically decreased after surgery.

**FIGURE 3:**
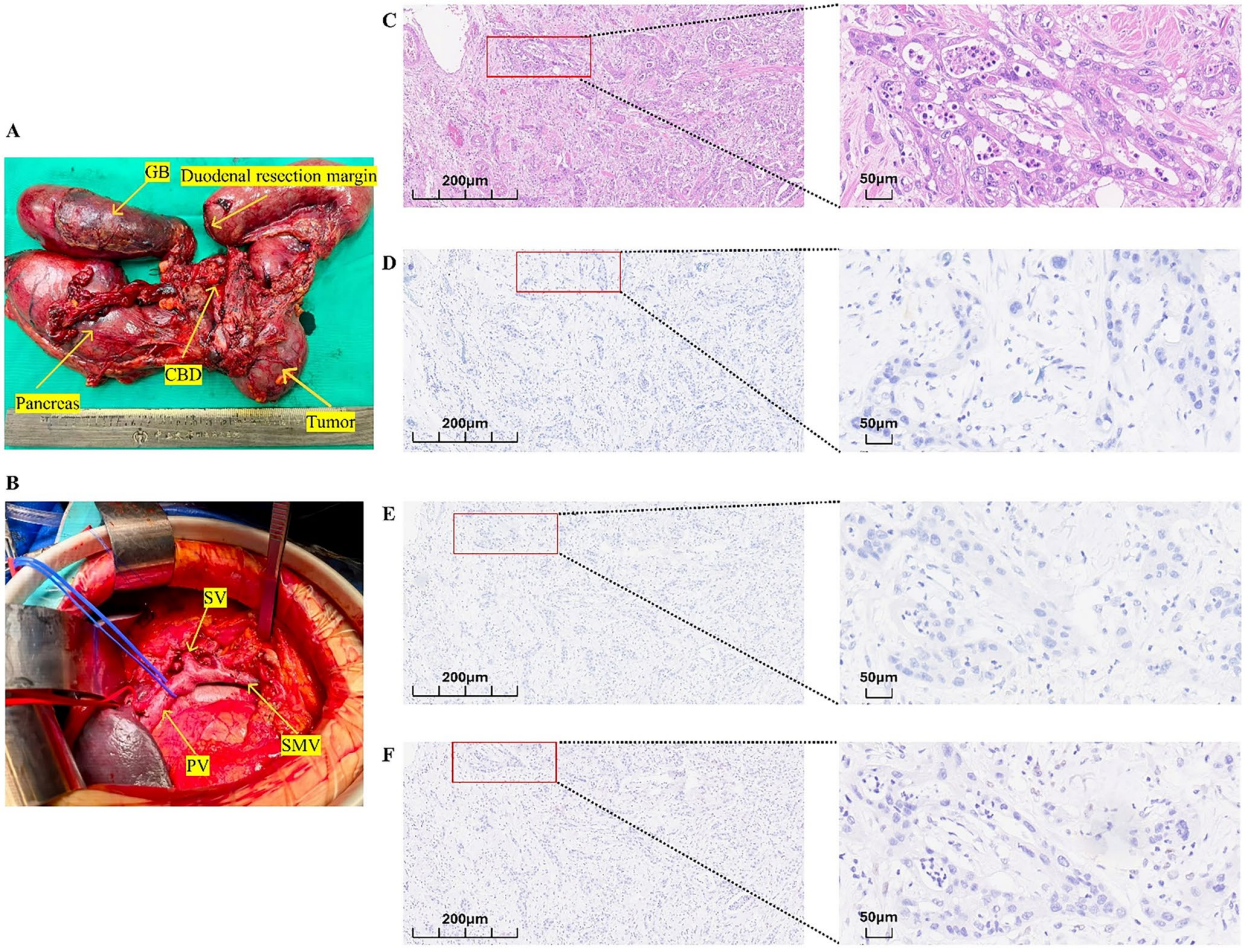
PDAC tumors and intraoperative vascular reconstruction. (A) Gross specimen, a tumor measuring approximately 4.0 × 5.0 cm was observed in the pancreatic head. (B) Intraoperative vascular reconstruction. The tumor invaded the SMV, and the involved vessel segment was resected, followed by end‐to‐end anastomosis. (C) HE staining shows that the pathological type of the tumor was pancreatic ductal adenocarcinoma (Left:×10, Right:×40). (D) AFP immunohistochemical staining is negative (Left:×10, Right:×40). (E) Glypican‐3 immunohistochemical staining is negative (Left:×10, Right:×40). (F) SALL4 immunohistochemical staining is positive (Left:×10, Right:×40).

We report a 67‐year‐old female pancreatic cancer patient with only elevated AFP before the clinical onset of PDAC, which was first detected in 2023 (Figure 1A) (Table 1). In April 2025, she was admitted to The Seventh Affiliated Hospital of Sun Yat‐sen University, presenting with abdominal distension, dull abdominal pain, jaundice, nausea, vomiting, and weight loss. Ultrasound demonstrated an occupying lesion in the pancreatic head, relatively well‐defined hypoechoic mass in the right upper abdomen with sparse vascularity and indistinct borders from the pancreatic head, and computed tomography (CT)/magnetic resonance imaging (MRI) identified a mass in the pancreatic head, which exerted compressive effects on the inferior vena cava and was associated with marked dilatation of the common bile duct, intrahepatic bile ducts, and pancreatic duct (Figure 1B,C). Furthermore, the AFP level of this patient was increased to 21.72 ng/mL (Reference range: 0–7 ng/mL) (Figure 2). In April 2025, after multi‐disciplinary treatment (MDT) discussion, the pancreaticoduodenectomy with partial resection of the superior mesenteric vein and vascular reconstruction was recommended (Figure 3A,B). Histopathology confirmed pancreatic ductal adenocarcinoma with negative surgical margins (R0 resection) and no lymph node metastasis (0/16). In May 2025, adjuvant chemotherapy with gemcitabine plus capecitabine was initiated 1 month after surgery. On the tenth day after the operation, the levels of AFP and CA19‐9 were decreased to 14.40 ng/mL (Figure 2) and 121.08 U/mL (Reference range: < 43 U/mL), separately.

**TABLE 1:** Timeline of clinical events.

Month/year	Events	Key findings/management
August 2023	Initial detection of elevated AFP (asymptomatic)	Contrast‐enhanced CT showed on evident lesions in the liver or pancreas. (Figure 1A)
April 2025	Symptom onset and hospital admission	The patient presented with abdominal distension and dull pain, jaundice, nausea/vomiting, and weight loss. Ultrasound showed a pancreatic head mass.
	Tumor markers	Serum AFP increased to **21.72 ng/mL** (reference: 0–7 ng/mL). Serum CA19‐9 increased to **946.22 U/mL** (reference: ≤ 43.00 U/mL).
	Preoperative CT	Contrast‐enhanced CT revealed a **~ 4.0 × 5.0 cm** mass in the pancreatic head with central non‐enhancing necrosis and peripheral ring‐like enhancement; gallbladder distension was noted; no hepatic lesions were identified. (Figure 1B)
	Preoperative MRI	MRI showed a pancreatic head lesion of **~4.8 × 4.8 cm**; no focal liver lesions; no obvious abnormalities of the portal venous system or hepatic vessels; gallbladder enlargement; marked dilatation of intra‐/extrahepatic bile ducts and pancreatic duct. (Figure 1C)
	Operation	Pancreaticoduodenectomy with partial SMV resection and vascular reconstruction was performed. (Figure 2A,B)
	Pathological diagnosis	Histopathology confirmed PDAC (Figure 2C); margins were negative (R0), and lymph nodes were negative (0/16).
Postoperative Day 10 (April 2025)	Early postoperative tumor markers	AFP decreased to **14.40 ng/mL**; CA19‐9 decreased from **946.22 U/ml** to **121.08** U/mL (reference: < 43 U/mL).
Postoperative ~1 month (May 2025)	Adjuvant therapy initiated	Adjuvant chemotherapy with gemcitabine plus capecitabine (GC regimen) was treated.
3‐month follow‐up (July 2025)	No recurrence	No evidence of recurrence was observed; AFP showed a gradual downward trend.
After 4 cycles of chemotherapy (Gemcitabine+Capecitabine) (August 2025)	Change the adjuvant therapy regimen	Recurrent diarrhea, weight loss, and intolerance to medication; switched to pembrolizumab plus oral trametinib.
7‐month follow‐up (November 2025)	Recurrence	Liver metastatic lesions were found
11‐month follow‐up (February 2026)	Death	Biliary tract infection, Obstructive jaundice, cachectic state

During chemotherapy with gemcitabine plus capecitabine, the patient presented with weight loss and recurrent diarrhea. However, there were no signs of recurrence or metastasis at 3 months after surgery, namely in July 2025. In August 2025, after four cycles of gemcitabine plus capecitabine chemotherapy, the regimen was subsequently switched to pembrolizumab combined with oral trametinib. At 7 months after surgery, in November 2025, liver metastatic lesions were detected, and fine‐needle aspiration confirmed PDAC. In February 2026, after 11 months postoperatively, the patient died of biliary tract infection and poor nutritional status.

## 3 Discussion

At present, pancreatic cancer surveillance depends on imaging modalities such as endoscopic ultrasonography (EUS), CT, and MRI, and reliable diagnostic biomarkers are still lacking [15]. CA19‐9 is the most established biomarker for PDAC, and is widely used to evaluate therapeutic response. However, its sensitivity and specificity significantly limit its value in clinical application [1, 16]. Especially, CA19‐9 exhibited poor ability in screening pancreatic cancer, with a low positive predictive value ranging from 0.5% to 0.9% [17]. Similarly, in the absence of CA19‐9 elevation, this case only presented with elevated AFP during the 2‐year asymptomatic period. Even so, literature reports indicated that compared with CA19‐9, the combination of CA19‐9/CA125 and CA19‐9/CEA could improve the sensitivity and specificity of pancreatic cancer detection, separately [18].

Interestingly, previous studies have indicated that many AFP‐producing tumor cells in PDAC show hepatoid or enteroblastic differentiation [19]. Herein, Hematoxylin–eosin (HE) staining confirmed PDAC rather than pancreatic ACC, and did not reveal hepatoid or enteroblastic differentiation. Meanwhile, Immunohistochemical staining showed AFP and glypican‐3 negativity, but SALL4 positivity (Figure 3C–F). Strikingly, our data showed that the AFP level decreased significantly after surgery. These findings supported the notion that the AFP level might be associated with the pancreatic lesion and/or the peri‐tumoral clinical status. Previous literature has reported that AFP‐producing pancreatic carcinoma with positive AFP immunohistochemistry, supporting the idea that pancreatic tumor cells could synthesize AFP [20]. In addition, AFP‐positive hepatoid carcinoma/hepatoid adenocarcinoma of the pancreas has also been reported, and these tumors may show AFP (and/or glypican‐3) positivity on immunohistochemistry [21, 22]. However, in our case, we think that negative AFP immunohistochemistry is not contradictory to positive serum AFP. First, AFP expression can be heterogeneous or focal in PDAC tumors, and sample bias may lead to negative staining if the AFP‐expressing component is limited or not included in the examined block. For example, hepatoid carcinoma of the pancreas has been reported with only focal AFP staining [23]. Second, we excluded hepatic inflammatory diseases and reproductive system tumors preoperatively, and the serum AFP level decreased after operation, which indirectly suggests that AFP originates from the PDAC tumors. Third, immunohistochemistry may have limited sensitivity, and serology–IHC discordance has been described in AFP‐producing gastrointestinal tumors [24].

Well known, AFP elevation is most commonly related to hepatocellular carcinoma and germ cell tumors [25]. Some findings also indicate that when a pancreatic mass is present together with elevated AFP, several pancreatic neoplasms should be considered. For example, hepatoid adenocarcinoma (HAC) and PDAC with enteroblastic differentiation, are clinically associated with elevated AFP and exhibit distinct hepatocellular‐like or enteroblastic features [19, 21, 26]. Furthermore, AFP elevation has been described as a recognized feature in pancreatic ACC and pancreatoblastoma (extremely rare in adults), which require differentiation based on characteristic morphology (e.g., squamoid nests) and acinar differentiation markers [10, 27, 28, 29]. Finally, although rare, pancreatic neuroendocrine neoplasms (pNET/NEC) may also produce AFP, particularly in progressive cases [6, 30]. Thus, comprehensive pathological evaluation and immunohistochemical staining are warranted to rule out these specific histological subtypes. In this case, the patient was confirmed to have PDAC, and there was no evidence of hepatoid/enteroblastic differentiation. And pancreatic HAC/ACC and other AFP‐producing pancreatic tumors were considered less likely.

Altogether, AFP is not a routine biomarker for PDAC, and its clinical utility in PDAC is still limited [13, 14]. However, AFP may have potential value in selected clinical scenarios. First, AFP could serve as an auxiliary clue to broaden the differential diagnosis and to consider rare AFP‐associated pancreatic tumors [21]. Second, serial AFP monitoring may be used as an individualized marker to reflect treatment response or disease progression [30]. Herein, It should be noted that we do not routinely measure AFP for pancreatic cancer patients. However, because this patient had a long‐term history of elevated AFP before admission, we performed serial AFP monitoring in this patient. We suggest that not all pancreatic cancer patients need to measure AFP. Instead, pancreatic‐origin lesions should also be considered in the patients with elevated AFP level after excluding liver lesions and genital system diseases.

Nowadays, there are no studies investigating AFP as a serum biomarker for pancreatic cancer, largely because AFP‐elevated cases of pancreatic cancer are exceedingly rare. Although AFP shows no advantage over other biomarkers such as CA19‐9 and CEA, pancreatic cancer should also be considered, and further endoscopic ultrasound (EUS), CT, or MRI detection could be warranted in these patients with the elevated AFP level after excluding liver cancer and reproductive system tumors. Pancreatic surveillance performed by EUS or MRI helps to detect small lesions, often less than 1.0 cm in diameter, which exhibit distinguishing features from the surrounding pancreatic parenchyma, such as hypoechogenicity and irregular margins [15]. And EUS has an advantage in histopathological evaluation. However, the diagnostic performance of EUS is highly operator‐dependent and closely related to the examiner's experience.

According to previous reports and the newest National Comprehensive Cancer Network (NCCN) guideline, the treatment strategies for pancreatic cancer patients with elevated AFP level are not different from those for patients with normal AFP level. Importantly, the therapeutic outcomes may be linked to factors such as tumor biological characteristics, vascular invasion, distant metastasis, and the patient's overall clinical condition [6, 27]. And previous studies indicated that postoperative adjuvant chemotherapy and R0 resection are independent prognostic factors for pancreatic cancer patients and significantly prolong survival [31]. Consistently, the patient underwent pancreaticoduodenectomy combined with superior mesenteric vein (SMV) resection(R0). In this case, although preoperative imaging did not reveal SMV involvement, our intraoperative findings showed the lateral wall of SMV was involved, with no evidence of intravascular tumor thrombus. Therefore, vascular resection and reconstruction were performed, and postoperative pathology confirmed R0 resection. Strikingly, the ESPAC‐3 trial showed no significant difference in overall survival (OS) between 5‐FU/leucovorin and gemcitabine, whereas data from the ESPAC‐4 trial supported the superiority of gemcitabine combined with capecitabine over gemcitabine monotherapy. Based on these findings and the NCCN guidelines, we subsequently selected the GC regimen for adjuvant treatment. After 3 months of standardized treatment, she showed no signs of tumor recurrence, accompanying with the gradually decreased AFP level [32, 33].

Interestingly, some studies have indicated that in some patients with portal vein (PV)/SMV vascular involvement, neoadjuvant therapy is associated with a survival benefit compared with upfront surgery [32, 33]. A phase III randomized PREOPANC trial from the Netherlands demonstrated that although the neoadjuvant chemoradiotherapy group achieved a higher R0 resection rate (71% vs. 40%) and a lower rate of local recurrence, upfront surgery showed several advantages: (1) avoidance of treatment delay, as 13% of patients in the neoadjuvant group experienced disease progression during treatment and consequently lost the opportunity for surgery. (2) reduced treatment‐related toxicity, since 52% of patients in the neoadjuvant chemoradiotherapy group experienced severe adverse events, and the rate of surgical complications was also higher (68.0% vs. 50.0%); and (3) avoidance of chemoradiotherapy‐induced pancreatic fibrosis, which increases surgical difficulty, as the overall resection rate was lower in the neoadjuvant group compared with the upfront surgery group (66% vs. 81.3%) [34, 35].

In conclusion, the distinctive feature of this case, compared with previously reported cases, the major novelty of the present study lies not simply in documenting another AFP‐elevated pancreatic tumor, but in showing that morphologically conventional PDAC, without overt hepatoid or enteroblastic differentiation and despite negative AFP/GPC3 immunostaining. More importantly, this patient presented with persistent serum AFP elevation and postoperative AFP decline. In contrast to the previously reported cases [13, 14], our case is distinguished by a prolonged preclinical phase of isolated AFP elevation before overt clinical presentation. Moreover, the literature has reported that AFP‐elevated gastric cancer is associated with a higher malignant potential and a poorer prognosis [36]. Based on the follow‐up data of this patient, it might support the notion that this specific PDAC with a high AFP level exhibits a more aggressive biological behavior and has a worse prognosis. In this case, the patient died 11 months after surgery because of tumor recurrence, cancer cachexia, and biliary tract infection. Therefore, for digestive system tumors with elevated AFP levels, more intensive postoperative follow‐up and surveillance may be warranted.

Overall, in the present case, AFP elevation persisted for 2 years, during which the patient remained asymptomatic. AFP may therefore represent a potential biomarker for pancreatic cancer, although this still requires further validation in future studies. Accordingly, when elevated AFP is detected, pancreatic cancer should also be considered after excluding hepatocellular carcinoma, germ cell tumors and gastrointestinal malignancies.

## Author Contributions

**Maoyun Xie:** conceptualization, methodology, writing – review and editing. **Qiang Tao:** writing – review and editing. **Xiaoying Wu:** methodology, resources, writing – review and editing. **Xiangling Lin:** methodology, resources, writing – review and editing. **Min Deng:** writing – review and editing, validation. **Wei Qin:** conceptualization, software, methodology, data curation, supervision, formal analysis, investigation, funding acquisition, writing – original draft, writing – review and editing, project administration. **Kaiwen Zhong:** software, data curation, formal analysis, writing – original draft, writing – review and editing. **Songlin Peng:** conceptualization, supervision, investigation, project administration.

## Funding

This work was supported by the Shenzhen Fundamental Research Program (JCYJ20220530145200001, JCYJ20240813150252047), the Medical Scientific Research Foundation of Guangdong Province (A2023384), Guangdong Basic and Applied Basic Research Foundation (2023A1515220088), and the Shenzhen Postdoctoral Research Fund.

## Ethics Statement

This study was approved by the ethics committee of the Seventh Affiliated Hospital of Sun Yat‐Sen University. The informed consent was obtained from the patient.

## Consent

Written informed consent was obtained from the patient for publication of this case report.

## Conflicts of Interest

The authors declare no conflicts of interest.

## Data Availability

The data that support the findings of this study are available on reasonable request from the author (Dr. Qin W, qw9911022@hotmail.com).
